# Healthcare Professional and Patient Perceptions of Changes in Colorectal Cancer Care Delivery During the COVID-19 Pandemic and Impact on Health Inequalities

**DOI:** 10.1177/10732748221114615

**Published:** 2022-08-20

**Authors:** Athena Ip, Georgia Black, Cecilia Vindrola-Padros, Claire Taylor, Sophie Otter, Madeleine Hewish, Afsana Bhuiya, Julie Callin, Angela Wong, Michael Machesney, James Green, Raymond Oliphant, Naomi J. Fulop, Cath Taylor, Katriina L. Whitaker

**Affiliations:** 1 School of Health Sciences, 3660 University of Surrey, Guildford, UK; 2 Department of Applied Health Research, 4919 University College London, London, UK; 3 Department of Targeted Intervention, 4919 University College London, London, UK; 4 Healthcare National Health Service (NHS) Trust, 3749 London North West University, Harrow, UK; 5 3661 Royal Surrey NHS Foundation Trust, Guildford, UK; 6 Hospitals NHS Foundation Trust, 8964 University College London, London, UK; 7 Patient Representative, UK; 8 9744 Bart’s Health NHS Trust, London, UK; 9 8930 NHS Highland, Inverness, UK

**Keywords:** colorectal cancer, qualitative research, inequalities, Covid-19, healthcare professional, pathway

## Abstract

**Background:**

The COVID-19 pandemic changed the way in which people were diagnosed and treated for cancer. We explored healthcare professional and patient perceptions of the main changes to colorectal cancer delivery during the COVID-19 pandemic and how they impacted on socioeconomic inequalities in care.

**Methods:**

In 2020, using a qualitative approach, we interviewed patients (n = 15) who accessed primary care with colorectal cancer symptoms and were referred for further investigations. In 2021, we interviewed a wide range of healthcare professionals (n = 30) across the cancer care pathway and gathered national and local documents/guidelines regarding changes in colorectal cancer care.

**Results:**

Changes with the potential to exacerbate inequalities in care, included: the move to remote consultations; changes in symptomatic triage, new COVID testing procedures/ways to access healthcare, changes in visitor policies and treatment (e.g., shorter course radiotherapy). Changes that improved patient access/convenience or the diagnostic process have the potential to reduce inequalities in care.

**Discussion:**

Changes in healthcare delivery during the COVID-19 pandemic have the ongoing potential to exacerbate existing health inequalities due to changes in how patients are triaged, changes to diagnostic and disease management processes, reduced social support available to patients and potential over-reliance on digital first approaches. We provide several recommendations to help mitigate these harms, whilst harnessing the gains.

## Introduction

Worldwide, the COVID-19 pandemic has had a devastating impact on the diagnosis and management of non-communicable diseases, including cancer.^1^ Rapid changes in healthcare delivery risked perpetuating existing inequalities in how people accessed and received medical care across the cancer care pathway.^2^

Delayed diagnosis is a continuing challenge because appointments or procedures (e.g., screening/endoscopy)^3^ are unavailable or a patient decides not to attend (e.g., due to fears of infection), healthcare systems are overwhelmed leading to changes in who provides care (e.g., clinicians with less experience in relevant field), and missed/delayed diagnosis because of reliance on remote consultations.^4^ Conceivably, these challenges may be more likely to happen in some groups, exacerbating existing health inequalities.

In cancer care, concerns were raised that changes in treatment options, as well as new institutional policies related to scarce resource allocation (e.g., *“reserving colonoscopy for those judged to be highest risk”*, p 17)^5^ led to a shift in focus from patient-centred to community well-being,^6^ that could unfairly disadvantage some patients. Conversely, rapid change has the opportunity to accelerate innovation, for example, by placing higher value on approaches with the greatest benefit such as the rapid adoption of a digital first approach^7,8^ and changes in policy, for example, the expansion of stool-based testing (faecal immunochemical test (FIT)) to triage people with colorectal symptoms.^9^

In the United Kingdom (UK), guidance for healthcare professionals (HCPs) was fast changing during the pandemic (e.g., the pausing and then reinstatement of endoscopy services)^10^ and was provided at national^11^ and local^5^ levels. This sometimes resulted in inconsistent messages,^12^ which may have influenced how guidance was enacted and interpreted by HCPs in different regions. Public facing messaging is also likely to have had an impact. For example, the message from the UK government to “stay home and protect the National Health Service (NHS)” and fears about being exposed to COVID-19 are thought to have led to people avoiding healthcare services.^13,14^ These changes, despite some being transitory, will provide important insights for future fast paced health system change, as well as focus on how more permanent changes (e.g., digital first) may impact on existing health inequalities.

We used colorectal cancer as an exemplar to draw lessons from changes in care delivery and consider how these changes may influence existing socioeconomic inequalities in cancer care. Using UK-wide interview data with patients and healthcare professionals and documentary analysis of documents detailing changes to cancer care during the COVID-19 pandemic, we sought to advance the evidence by answering the following questions: (1) what were the main changes in colorectal cancer care delivery during the pandemic? (2) how were these communicated by policy and guidance documents, and received and interpreted by HCPs? (3) what was the impact of these changes on patients, particularly on inequalities in care?

## Methods

### Approach

Policy and guidance documents about adapting colorectal cancer delivery were triangulated with semi-structured interviews with HCPs and patients to understand the main changes to the colorectal cancer pathway during the COVID-19 pandemic and impact of these changes on socio-economic inequalities.

### Participant Selection and Recruitment

Patients were recruited through a research company (Saros) and a screener was used to identify people from higher and lower socioeconomic groups (indexed by education) across the UK who had experienced symptoms related to colorectal cancer during the pandemic and sought medical help.

HCPs working across the colorectal pathway were recruited across the UK using a snowballing technique, whereby our research advisory group consisting of health professionals, researchers and patients shared the study information to eligible people who might be interested in taking part. We continued to snowball through HCPs we interviewed.

Both patients and HCPs provided verbal informed consent to participate. Sample size and choice of SES index/categories were derived from our previous qualitative research with patients^15^ and healthcare professionals^16^ and based on norms for qualitative research using purposive sampling.^17^

### Data Collection

#### Patient Interviews

Semi-structured interviews with patients were carried out by an experienced qualitative researcher (AI) from October-November 2020 via phone or Zoom (mean duration = 59 minutes; range: 31-86 minutes). Follow-up interviews via phone or Zoom were also carried out with some patients who reported ongoing interactions and had not already spoken about their experience of further investigations in their initial interview (mean duration = 16 minutes; range: 8-23 minutes). In a previous paper^18^ we focused on patient accounts of accessing primary care. In this paper, we focus on patients who were referred to secondary care for investigations.

#### Health Care Professional Interviews

Semi-structured interviews with HCPs were carried out by AI between February 2021 and August 2021. Interviews took place via phone or Zoom and lasted on average 30 minutes (range: 17-50 minutes). Interviews focused on understanding HCPs’ views of the main changes to colorectal cancer care delivery during the COVID-19 pandemic, their perceptions regarding if and how these changes impacted socioeconomic inequalities. Please see Supplementary Material 1 for the HCP topic guide. The patient topic guide has been previously published.^18^

#### Document Gathering

Document gathering on changes to cancer care during the COVID-19 pandemic in both primary and secondary care took place throughout the study by engaging with professional bodies such as NHS England and Improvement and the Association of Coloproctology of Great Britain and Ireland.

### Analysis

We used Rapid Assessment Procedure (RAP) sheets alongside interviews to synthesise and gather real time insight into the data prior to transcription. RAP sheets are a tool used in rapid qualitative research to summarise findings and share them in a timely way,^19^ which is particularly important when aiming to produce actionable findings.^20^ We used the candidacy framework as an analytical tool^21^ to understand changes in how people accessed/received healthcare. After professional transcription, transcripts were repeatedly read by AI and quotes were drawn out to provide a more in-depth analysis of the data in the RAP sheets. AI along with three members of the research team (KW, GB, CV) had multiple data analysis meetings to further refine the findings and ensure that the final themes reflected the data. These were then further discussed with the wider research team consisting of HCPs and patient representatives.

#### Patient Interviews

Comparative thematic analysis was carried out to explore differences between higher and lower SES groups, which involved first conducting analyses on each group separately, before moving to analyse differences between the groups.

#### Healthcare Professional Interviews

Two RAP sheets were developed to explore changes in the diagnostic pathway and the treatment pathway. The diagnostic pathway included data from professions involved in pre-diagnosis (e.g., General Practitioners (GPs), gastroenterologists). The treatment pathway included data from professions working with patients post-diagnosis (e.g., Clinical Nurse Specialists).

#### Document Analysis

Retrieved policy and guidance documents were summarised for content, particularly looking for aspects that could relate to inequalities either directly or indirectly. These extracts were triangulated with the rest of the dataset, comparing document and interview data about specific changes in the pathway.

## Results

### Patients

Of the 39 patients initially interviewed, fifteen (38%) reported referrals to specialists for further investigations (e.g., scans) or treatment and four out of the fifteen patients had a follow-up interview as they reported ongoing interactions. We present findings from all 39 participants (focused on primary care experiences) in another paper.^18^ Out of the 15 patients, 6 were from lower socioeconomic backgrounds and 9 were from higher socioeconomic backgrounds. The average age of this sub-sample was 57 years and 60% were female. Most were from White ethnic backgrounds (n = 12), one participant identified as Black African/Caribbean, one as Indian and one as Asian/Asian British. None of the patients disclosed a diagnosis of cancer during their interviews.

### Healthcare Professionals

A total of 30 HCPs were interviewed across rural and urban areas of England, Wales and Scotland. The sample consisted of GPs (n = 5), specialist screening practitioners (n = 5), radiologists (n = 2), gastroenterologists (n = 2), oncology pharmacists (n = 3), clinical nurse specialists (n = 3), surgeons (n = 5) and oncologists (n = 5). See Table 1 for demographics.

**Table 1.** Healthcare Professional (HCP) Demographic Characteristics.

Ethnicity	N	%
English/Welsh/Scottish/Northern Irish/British	20	66.7
Any other white background	2	6.7
Irish	2	6.7
Chinese	2	6.7
Mixed/multiple ethnic groups	1	3.3
Indian	3	10
Sex		
Female	18	60
Male	12	40
Type of setting		
Primary	5	16.7
Secondary	22	73.3
Tertiary	3	10
Years qualified as HCP		
≤10	3	10
11 to 20	14	46.7
21-30	10	33.3
31-40	3	10
Years working with colorectal cancer patients		
≤10	16	53.3
11 to 20	10	33.3
21-30	4	13.3

Table 2 summarises the main changes in colorectal care and potential impact on inequalities. We also present a timeline of key events and policies as supplementary material (see Supplementary Material 2).

**Table 2.** Summary of Main Changes in Colorectal Care and Impact on Inequalities.

System Change in Colorectal Cancer Care	Policy References	Challenges with Implications for Inequalities in Care	Potential Improvements for Access and Equality of Care
Use remote (e.g., telephone, video, email etc) technology as first point of contact	NHS England and improvement: • *Letter to primary and secondary care from NHS leaders*^22^	• Not everyone is comfortable using remote technology	• Improved access/convenience
	• *Primary Care Cancer Referral Guidance in Response to COVID-19*^23^	• Not everyone has access to video technology	• Less expense and time incurred by patient not traveling
	• *Briefing template to cancer alliances*^24^	• Remote clinician contacts are often not attached to fixed times, which increases uncertainty when the consultation to occur	• Removes challenges around competing priorities (e.g.,, Work/childcare)
		• Challenges with involving interpreters remotely and use of informal (family/friends) rather than professional interpreters	
Use faecal immunochemical test (FIT) to triage symptomatic patients	NHS England and improvement, clinical guide for the management of patients requiring endoscopy during the coronavirus pandemic^25^	• Differences in ease of completing test/following instructions for both primary care and patient	• Improved triage/prioritising process
	The Association of Coloproctology of Great Britain and Ireland. *Considerations for adapting the rapid access colorectal cancer pathway during COVID-19 pandemic*^26^	• Challenges in implementing this in primary care	
	Local guidance: • (e.g.,) Healthy London Partnership: *Primary Care FAQ - Lower GI 2WW pathway during the COVID-19 Pandemic*^27^	• Challenges in secondary care having access to FIT tests	
	• Northern cancer Alliance. q*FIT (quantitative faecal immunochemical test) in Primary Care for - Lower GI 2WW pathway FAQs*^28^	• Increased complexity in navigating tests/sites	
Create COVID-free hospitals/ring-fence facilities	NHS England and NHS improvement: • *Advice on maintaining cancer treatment* during the COVID-19 response^29^	• Impact on requirements on patients and family re procedure/treatment COVID infection control policies	• Improved convenience
	• Advice on maintaining cancer recovery^30^	• Discrepancies in how/if patients were given information (e.g.,, no news is good news)	
	• Standard operating procedures for infection prevention and control.^31,32^	• Increased fear of attending hospitals due to infection risk	
Limit visiting by family/friends, maintain careful visiting policies	• NHS guidance on visiting someone in hospital^33^	• Challenges in arranging childcare to attend appointments	• Use of professional rather than informal interpreters
		• Lack of advocacy, limiting shared decision making and reducing emotional support	
Adapt treatment pathways/prioritise care to reduce number of patients attending hospital/limit patient exposure to COVID-19	Adaptation of existing treatment pathways,^29-31,34-36^ including: • Shorter course radiotherapy^37^	• Increased onus on patients to advocate and manage for themselves in new settings (e.g.,, from home)	• Improved access/convenience
	• Use of oral medications at home	• Responsibility of HCPs to prioritise care for those most likely to benefit	• Removes challenges around competing priorities (e.g.,, Work/childcare)
	• Suspension of all non-urgent surgery^38^		

### Use of Remote Technology

On March 17^th^ 2020, healthcare providers were told by the NHS England to roll out remote consultations to the most vulnerable in the first instance and then for other patients.^22^ Video was indicated as the preferred format for remote consultations in secondary care as it was suggested that this would allow for better clinical judgement and communication when managing patient expectations and discussing relative risks and benefits.^26^

HCPs described the positive impact of the move to remote technology on inequalities:

“I think there’s no doubt that a huge number of patients have benefitted from video consultations and telephone consultations and not having to come to the hospital, particularly those who are working and have got childcare duties it can greatly improve their quality of life by not having to come and sit in a hospital waiting room.” (P13, Gastroenterologist)

Although it was recognised that there would be differences in whether people had equitable access to technology:

“I mean maybe if you’re doing more virtual clinics maybe you know, we’re phoning them so they’re not paying for the calls, but do they have the kit for the virtual clinic, does everyone have a tablet or an iPad you know, maybe they do, maybe they don’t, that might be one inequality.” (P1, Colorectal Surgeon)

#### Practical Challenges With Video Options

Despite video being recommended, patients were reticent about using video calls and were more likely to choose the telephone option:

“With the video it’s not so bad but very few people are taking up the video option, most are going for the telephone option and of course not everybody has the technology. Whilst you do the best you can with a telephone consult, it’s not quite the same as a face-to-face.” (P26, Specialist Screening Practitioner)

There were issues with video access on the healthcare provider side too, as one HCP mentioned issues with technological capability in the hospital:

“I think the guidance would have been geared more towards video consultations and I think that we haven’t been able to really put that in place just due to technological issues and problems with the internet and intranet facilities. So we, I’m doing this [interview] on my own laptop because sometimes the laptops and the bandwidth of the hospital internet is not as good.” (P13, Gastroenterologist)

#### Challenges With Involving Professional Interpreters Remotely

Another challenge was the remote involvement of interpreting services and the impact this would have on some patients (for example with limited English proficiency). This was both an issue of supply as well as quality of care, as the limited number of translators available were unable to replicate the detail of face-to-face consultations on the telephone:

“Although we could use the interpreter service via the telephone, again, you lose that aspect of the person actually, that physical aspect of being able to see the person has understood what has been put to them through the interpreter. Because at least when you can see somebody and they’re smiling, you can see it in their face that they do understand what you’ve said or what has been interpreted to them.” (P26, Specialist Screening Practitioner)

This led to reliance on some practices that are not recommended (because they are related to worse patient outcomes), such as using family or friends as informal interpreters:

“Our hospital policy is we don’t use family, we should use hospital translators, but because of the pandemic we had to, you know, kind of compromise and not do that.” (P19, Specialist Screening Practitioner)

#### Unforeseen Issues Navigating Digital Care

Patients with ongoing interactions with the healthcare system also highlighted the need to provide flexibility. They felt reliance on remote consulting would lead to gaps in care and exacerbate existing challenges with navigating the system and getting the right medical support:

“I know there are people that find it really difficult to kind of access healthcare services anyway, because of their own kind of social barriers […], and if that was made more difficult by, for example, a telephone consultation where a doctor called them back and if they missed the call they'd missed the consultation, that kind of thing could make it quite difficult for some people to get the help they needed.” (Lower SES, P36)

In summary, the introduction of a telephone first approach has potential benefits related to ease of access/permeability of services because having an appointment by telephone or video call does not involve travelling or managing other competing priorities. However, this shift may present challenges because not everyone has access to the same technology or feels comfortable using it. Remote consultations also presented new practical challenges in terms of ensuring availability when a HCP calls and organising access to formal interpreting services remotely. Patients and HCPs expressed concern that these new challenges may impact how people appear at services (e.g., convey their symptoms) and adjudicate for themselves (e.g., asking for the help they need, or demonstrating understanding of ongoing care/referrals).

### Use of Faecal Immunochemical Test to Triage Symptomatic Patients

In response to the diagnostic capacity issues because of the COVID-19 pandemic, the FIT test was implemented in some regions of the UK to triage symptomatic patients on the 2 weeks wait pathway for fast track cancer referrals.^27,28^ In London, changes to the pathway were made mandatory in June 2020, requiring all patients with symptoms suggestive of possible colorectal cancer to have a FIT test before being referred to secondary care unless they had a rectal or anal mass, or anal ulceration.^27^ On 9^th^ April 2020 guidance was proposed for adapting the rapid access colorectal cancer pathway during the COVID-19 pandemic to include FIT testing in primary care.^26^

The introduction of FIT test kit for triage was welcomed by clinicians but also highlighted as a potential area where inequalities could be perpetuated:

“I think while I understand the, the necessary introduction of that part of the pathway, that, that’s not an easy test to produce for, for some people who have, who maybe that, the elderly, the frail, those with learning difficulties, mental health, so the FIT test I think in some ways increases inequalities because those patients who haven’t done a FIT test may go to the bottom of the pile, bottom of the queue to, to see the speciality team.” (P6, GP)

From the patient perspective, only participants in the higher SES groups mentioned completing the FIT test (n = 3) as part of their ongoing interaction with healthcare and described it as a straightforward, fast-tracked process:

“Well he got to it very quickly, he was able to get me to go and do a test really fast, he didn’t, there was not this hesitation like oh you know, this is not a priority, we’ll wait, there was no waiting.” (P27, higher SES)

But completing this step in the triage process also involved additional actions on the participant, for example, to chase for the results:

“It all came through the post as a kit, I did it and returned it within a couple of days I think. But I sat there and waited and waited, so after about 6 weeks I rang the consultant’s secretary, she said she’d chase it along, and I repeated that 2 weeks later and then I got a letter from the consultant just to say it showed no abnormalities.” (P28, higher SES).

The rapid introduction of FIT is an innovation that has the potential to improve the diagnosis of colorectal cancer. However, this additional step in the process may impact inequalities because it reduces the ease with which people can use services because an additional demand/threshold is required before moving on to the next step of the care pathway.

### Create Covid-free Hospitals by Ring-Fencing Facilities

On March 17^th^ 2020, elective surgery was delayed and only urgent or essential surgery continued.^22^ The NHS released advice on 30^th^ March 2020 to help maintain cancer services and recommended that regional offices and local systems develop plans for cancer and consider the consolidation of cancer surgery on ‘clean sites’, as well as COVID testing 48 hours before surgery.^29^

HCPs noted several practical challenges with COVID infection control measures, such as the requirement to self-isolate and differences in how people may respond to this and their awareness of alternative options:

“The main hospital that I cover are still requiring patients to have a PCR test and isolate for 3 days, so we are giving patients the option, if they are saying I can’t isolate for 3 days because I’m working, I’ve got childcare issues, then we do offer them to attend one of the other hospitals which is only offering the lateral flow swabs, so they wouldn’t need to isolate for the 3 days.” (P29, Specialist Screening Practitioner)

Another aspect that was highlighted in relation to inequalities was the requirement for patients to navigate to different sites that they may have been less familiar with:

“Since the pandemic all the clinics have moved to another hospital in our Trust, but it’s a different location so that was a big thing both for staff and patients because it was new thing, you know, we weren’t able to do the clinics on site and the operations.” (P19, Specialist Screening Practitioner)

Patients described procedures such as undertaking a COVID PCR test before their hospital appointment, and higher SES participants seemed to view this positively, experiencing few concerns or difficulties despite increased complexity in the process:

“Prior to going to the biopsy appointment, I was informed that I needed to attend an outdoor clinic for a swab test to make sure that I was negative for COVID-19 otherwise the appointment for the biopsy would have to be cancelled. I attended for the COVID test on a Sunday morning, and I received a telephone call on the following Tuesday morning confirming that it was negative and I could continue with my appointment to have a biopsy.” (Higher SES, P32)

In contrast, fear of hospitals and lack of trust in mitigation procedures were more apparent in reports from lower SES participants:

*“*It is not good enough, I need a 100% clarification that I absolutely positively do need to go, before going to a plague house.*”* (Lower SES, P13).

This may lead to some people being more likely to resist services than others. These changes in the system present another example of how the service may have become less permeable for some due to the increased complexity in the pathway, as well as new demands in terms of being aware of services, navigating and utilising them.

### Limit Visiting by Family and Friends, Maintain Careful Visiting Policies

Limitations on visiting policies varied across different hospitals and included suspending or limiting the number of people visiting to reduce the spread of COVID-19.^33^ The impact of these restrictions on inequalities was highlighted by HCPs in terms of managing childcare, or processing information and feeling confident in accessing care:

“Before you can maybe bring your kids if you had no daycare or if you needed or you’re a bit forgetful, you know, you can have somebody come with you, but now we wouldn’t obviously, that wouldn’t be allowed.” (P7, Radiologist)

And this was echoed by patients:

“I think, had there not been such a rush, had the Covid not been in place, may have allowed things like someone to come and stay with me or be with me, not necessarily during surgery, but certainly in the recovery period [….] I wasn’t always able to describe how my feelings were and I feel like a lot of that sort of anxiousness and that sort of deeper thought those difficulties could have been avoided with a bit of extra support.” (Lower SES, P22)

These considerations are important because they highlight the contextual nature of people accessing healthcare services amongst their own competing life/work priorities, as well as the importance of having people there to advocate for them when presenting at/using services.

### Adapt Treatment Pathways and Prioritise Care to Reduce Number of Patients Attending Hospital, and Limit Patient Exposure to COVID-19

Adaptions to treatment pathways and the way in which treatment was prioritised was highlighted by HCPs as important, including the offer of shorter course radiotherapy, oral medications delivered to people’s homes, and new ways to prioritise care. There was discussion around a reduced burden on patients, which was perceived as a positive impact on care:

“Certainly, some of the things that I would look at traditionally around access to treatment so can you get yourself to the hospital, some of that has been circumvented because of the processes that we’ve put into place with COVID so we’re making phone calls, telephone clinics to patients, we’re also delivering medicine to patients who are on oral treatments. I would guess that that may, proportionally speaking, benefit people of lower socio-economic status in a positive way than people of a higher socio-economic status, but that would just be my gut feeling.” (P10, Oncology Pharmacist)

Although there was also concern about exacerbation of inequalities because patients were required to self-advocate (for example, during remote consultations, or in person but without support of family/friends), which was perceived as easier for those in more affluent groups:

“I mean patients sometimes have to push for themselves to get, they don’t get forgotten as such because they’re all you know, but sometimes say a scan’s not been booked because there’s less scans happening because the staff aren’t there […], they’ve been redeployed or, so if a patient’s not pushy enough sometimes. I don’t know, would a patient stand up for themselves more if they were sort of better educated and knew what they should be having.” (P3, Clinical Nurse Specialist)

“I would argue that the people with less resources, either material resources or social resources, would have more difficulty navigating those different processes than, you know, your more affluent, sharp elbowed middle-classes, because part of the challenge when there is a big system change is that it takes a lot of work to navigate.” (P22, GP)

Interviews with HCPs also revealed increased responsibility and pressure on them as professionals to prioritise care during a time of limited resources, where there is also an additional risk that socially disadvantaged groups may be perceived as less eligible for services due to factors such as relatively poorer potential fitness for treatment:

“We had to be careful about case selection and certainly that's what we adopted here […] You had to select your patients I would say in that we tended to go for the people who were physically fit, you know, very physically fit, because you had to be sure that if they came in for an operation they would get up and walk away five or six days later, because if we had major complications it would consume an ITU bed for a length of time. I know that sounds quite cynical and harsh, but it was the only way that it could work.” (P5, Colorectal surgeon).

In this theme, practical benefits of ease and convenience of receiving treatment at home were weighed against novel challenges. For example, patients needed to navigate new ways to communicate with HCPs with reduced contact or in person visits. Patients and HCPs’ adjudication for care in the new colorectal care pathways were susceptible to exacerbation of inequalities due to the new thresholds for eligibility. This included physical fitness but also an increased role for patient self-advocacy and navigation.

## Discussion

This study identified main changes in colorectal cancer care delivery during the pandemic and tracked how these were implemented through policy and guidance documents. We reported how these were received and interpreted by HCPs and considered the impact on inequalities in care for patients. HCPs reported rapid, transformative change that provided ways in which services could be delivered safely and were more accessible and convenient for patients, as well as helping to prioritise those most in need. However, there were also unintended consequences of these system changes.

Four main areas of change were highlighted that could pose ongoing problems in terms of exacerbating inequalities in care, despite applying to all patient groups: use of/reliance on remote technology, introduction of FIT into the colorectal cancer pathway, creating COVID-free hospitals (including changes in visitation policies), and changes in treatment pathways. HCPs accounts, supported by data from patients, showed that there was concern that changes in the way patients presented at/navigated services, discussed their health concerns and received advice/follow-up care were likely to disadvantage some groups more than others, particularly those facing competing priorities, as well as those less able to advocate for themselves.

### Comparison With Previous Research

The concept of ‘candidacy’, which describes how people’s eligibility for healthcare is determined between themselves and health services,^21^ helps explain our findings. The move to remote consultations was beneficial for convenience, but raised new challenges related to how people presented at services, such has potential loss of information and prompts from not being able to see people in person (or via video link), challenges involving remote interpreters, and uncertainty about when consultations would happen, or how to follow-up remotely. HCPs were concerned that these unintended consequences may be more likely to impact people from lower socioeconomic groups and this was echoed by patients. Concerns around digital technology exacerbating inequalities were raised pre-pandemic^39^ and have been raised several times since.^7,40,41^ Reasons for differences in being able to benefit from the “digital boom” include not having internet access/technology, lack of private space and differences in skills to engage with remote consultations.^40^

The introduction of FIT into the colorectal cancer pathway was welcomed by clinicians as an improvement to the diagnostic pathway. Recent evidence suggests that FIT is as sensitive at selecting patients with suspected colorectal cancer symptoms for urgent investigation irrespective of socio-demographic characteristics such as deprivation status.^42^ However, our findings reveal HCPs also expressed concern that some people may be more likely to complete the test than others, leading to inequality.

System changes related to making hospitals safe such as COVID-19 testing protocols, ring-fencing certain hospitals and restrictions on visitation policies are also likely to impact inequalities. They highlight the importance of people’s ability/desire to access (e.g., issues related to transport/practical implications of accessing hospitals on different sites), as well as use services (e.g., we saw evidence that some patients decided they would not utilise a health service due to fear of infection risk). Evidence during the pandemic found that South Asian adults were less positive towards measures to reduce hospital-based COVID-19 transmission during colonoscopy than White adults.^43^ In addition, perceived constraints to access healthcare have been shown to vary by socio-demographic characteristics, such as personal mobility,^44^ and differences in patient behaviours (e.g., help-seeking) have already been mooted as an explanation for inequalities in late presentation of colorectal cancer.^45^

Finally, changes in treatment pathways also created challenges for inequalities such as new ways to prioritise care or new ways of administering treatment. In a study exploring global changes to chemotherapy service during the pandemic, nearly half of institutions surveyed reported implementing treatment prioritisation strategies where treatment was postponed, reduced or stopped for some patients.^46^ Evidence already shows that lower SES groups are less likely to receive treatment for colorectal cancer^47^ and therefore this is another potential area where inequalities may be perpetuated.

### Practical Implications/Recommendations

We saw very little discussion of inequalities in the documents we gathered during this yearlong study. In addition, national priorities for health inequalities post-pandemic are often quite broad (e.g., *“bring questioning and challenge to ensure health equity is at the heart of plans for restoring services”*)*.*^48^ Our research highlights specific actions that could help directly address the risk areas highlighted by clinicians across the care pathway.

One recommendation is to support and build on training for staff to address inequalities, particularly around access and to support people with barriers to digital inclusion. Clinicians and commissioners need to be provided with up to date, evidence-based guidance on best practice for remote interpreting services for different populations experiencing language barriers, such as people with limited spoken English language proficiency.^49^ Other innovations, such as the use of FIT in the pathway also need to be tracked/audited to anticipate challenges in using the test from both the patient and HCP perspective.

Our findings showed challenges for patients in terms of navigating services that also need addressing. For example, approaches aimed at ensuring patients understand how to access and utilise care may be more important than ever, given the potential for exacerbation in inequalities highlighted here, as well as recent evidence demonstrating that low health literacy (e.g., understanding health information) is associated with longer primary care intervals, impacting on timely cancer diagnosis.^50^ One recommendation is that clinicians use an approach called health literacy universal precautions, which assumes that most patients may be at risk of mis-interpreting health information by keeping communication simple and checking understanding.^51,52^ Patient navigators could also play an important role in adjudicating for patients and improving timely cancer care.^53^

### Strengths and Limitations

We have triangulated data across policy documentation, patient interviews and interviews with HCPs to provide unique, in depth insights into how inequalities in cancer care may be perpetuated across the care pathway and provide recommendations for action. A strength of this study was that by gathering policy documentation prior and during the interviews we were able to provide relevant prompts and ensure that we captured views on the main changes as the pandemic occurred, rather than ask people retrospectively to reflect on these changes.

This study included a varied sample of patients and HCPs across the UK from different regions of England, Scotland, and Wales and drew on documentation from all UK nations. We recruited our sample using a snowballing method through our advisory team. This method of sampling may have led to oversampling from a particular region of the UK however, due to the variation in our team/final sample we are confident that we were able to capture diverse views. This study was conducted from October 2020 to early in 2021 and does not necessarily capture changes that happened later in the pandemic. We used a market research company to recruit patients based on the rapid need to understand changes in real time, but this may have had limitations in terms of representing the views of those who may be less likely to engage with research.

We focused on diagnostic and treatment phases of the cancer care pathway, and it is important for future research to understand how the COVID-19 pandemic may have exacerbated inequalities for people at different points in the care pathway, for example, those living with and beyond cancer, as well as those receiving palliative care.

## Conclusion

The COVID-19 pandemic caused drastic changes to the healthcare system and our research suggests that some of these changes may have had a positive impact, whilst others may have exacerbated existing inequalities in cancer care. Recommendations are provided to help minimise these impacts during and post-COVID-19, and also highlight areas to be aware of in the event of future pandemic(s). These recommendations are also likely to have relevance beyond colorectal cancer.

## Supplemental Material

Supplemental Material - Healthcare Professional and Patient Perceptions of Changes in Colorectal Cancer Care Delivery During the COVID-19 Pandemic and Impact on Health Inequalities Click here for additional data file. Supplemental Material for Healthcare Professional and Patient Perceptions of Changes in Colorectal Cancer Care Delivery During the COVID-19 Pandemic and Impact on Health Inequalities by Athena Ip, Georgia Black, Cecilia Vindrola-Padros, Claire Taylor, Sophie Otter, Madeleine Hewish, Afsana Bhuiya, Julie Callin, Angela Wong, Michael Machesney, James Green, Raymond Oliphant, Naomi J. Fulop, Cath Taylor, and Katriina L. Whitaker in Cancer Control
