# Supplementary material for: Healthcare Professional and Patient Perceptions of Changes in Colorectal Cancer Care Delivery During the COVID-19 Pandemic and Impact on Health Inequalities
Source: Cancer Control. 2022 Aug 20;29:10732748221114615. doi: 10.1177/10732748221114615 (PMC9393398; doi:10.1177/10732748221114615)
Supplement: Supplemental Material - Healthcare Professional and Patient Perceptions of Changes in Colorectal Cancer Care Delivery During the COVID-19 Pandemic and Impact on Health Inequalities [file sj-pdf-1-ccx-10.1177_10732748221114615.pdf]

Supplementary Material 1

Topic guide for project "**COlorectal cancer and COVID**"

[Introduce yourself – check for questions based on PIS – check confidentiality understanding – take formal consent-check about audio recording]

- *Trying to understand healthcare professional views of the main changes to colorectal care delivery during COVID-19*
- *Also interested in how these changes will impact socioeconomic inequalities in cancer care*
- *Interested in what you perceive to be "optimal" practice in terms of lessons learnt, changes going forward and how sustainable these are*

- To start with, if you think about the pathway for colorectal care delivery during the pandemic and you think about the pathway pre-pandemic what are the main differences?

- What types of guidance or policy did you follow to make changes? What do you think about these? (e.g. where do you get your information? Interested to know how you made changes)

Example prompts

- What have you learnt from these changes?

- When patients come to visit your service, what do they encounter?

- How do you think these changes have affected patients' experiences of care?

- Do you think these changes may impact some groups more than others? (e.g. people from more or less deprived backgrounds) Why is this the case?

- What aspects of the pathway do you think could be adapted to make it easier for people with different backgrounds?

- What adaptations were made to the pathway to make it easier for people with different backgrounds if any? Was there a gap between what you wanted to do and what you did do?

- If another situation like this pandemic were to happen, what changes do you think shouldn't be kept? Why not?

- What changes do you think should be kept? What are your thoughts about adopting these changes in a post-covid era? If not, why not? How sustainable do you think these changes are?

If noone mentions workforce issues (offsick or being redeployed):

- Also interested in whether staff workforce issues played a role for you? How did that make things more challenging and how did that have an impact on inequalities?

Recorder off

Supplementary Material 2

Colorectal cancer pathway changes

TIMELINE OF KEY EVENTS AND POLICIES

2020

JANUARY 30th
First phase NHS response

Mar

MARCH 16TH
NHS visiting restrictions limited at some hospitals

MARCH 17TH
Surgery delayed and urgent only, modification of existing cancer waiting time guidance. Use of telephone triage and telephone as first appointment. Home delivery of oral treatment.

MARCH 25TH
FIT + bowel screening endoscopy paused (6 weeks).

MARCH 26th
UK first lockdown

MARCH 30TH
Urgent and essential cancer surgery at 'clean sites' and COVID testing 48 hours before surgery

Apr

APRIL 8TH
Suspension of visiting

APRIL 16TH
Lockdown extended. Government sets out five tests that must be met before restrictions are eased.

APRIL 29th
Second phase NHS response

APRIL 29TH
Recovery of surgical services.

APRIL 30TH
Guidance on Endoscopy early recovery and testing before procedures.

May

MAY 10TH
Prime minister announces conditional plan for lifting lockdown: people who cannot work from home should return to the workplace but avoid public transport

Jun

JUNE 1ST
Phased re-opening of schools in England

JUNE 15TH
Non-essential shops reopen in England

JUNE 23RD
PM says UK's "national hibernation" coming to an end – announces relaxing of restrictions and 2m social distancing rule

Jul

Aug

AUGUST 1ST
Restore full op of all cancer services cancer alliance to draw up delivery plans for sept 2020 to March 2021

AUGUST 1st
Third phase NHS response

Sep

Oct

Nov

NOVEMBER 5th
UK second lockdown

Dec

DECEMBER 2ND
Second lockdown ends after four weeks and England returns to a stricter three-tier system of restrictions

DECEMBER 8TH
First COVID vaccine in UK

DECEMBER 15TH
Testing for COVID before hospital procedures

DECEMBER 21ST
Tier 4 restrictions come into force in London and South East England

2021

Jan

JANUARY 6th
UK third lockdown

JANUARY 21ST
Guidance for maintaining services within health and care settings: infection prevention and control (IPC) recommendations'

Feb

FEBRUARY 24TH
First interview

AUGUST 6TH
Last interview
